# Carbimazole Embryopathy and Choanal Atresia

**Published:** 2014-01-01

**Authors:** V Raveenthiran

**Affiliations:** Department of Pediatric Surgery, SRM Medical College and Hospital SRM University, Chennai, India.

Congenital choanal atresia (CCA), especially the bilateral form, is a rare neonatal emergency. Its incidence is usually quoted as 1 in 5000 - 8000 live-births. [1] However, a recent epidemiological study involving more than 2.2 million deliveries suggests that CCA is extremely rare with a calculated incidence of 3 to 9 per 100,000 live-births. [2] Athena is fascinated by CCA not only due to its rarity but also by the dramatic nature of symptoms. Refusal to suckle on ipsilateral breast of mother is pathognomonic of unilateral CCA while cyclical breathing (temporary relief of cyanosis by crying) is characteristic of bilateral disease. Occasionally, CCA may be a component of an established syndrome such as CHARGE association and Antley-Bixler syndrome. [1] Nevertheless, CCA is predominantly a sporadic malformation with no genetic predisposition. Etiology of the non-syndromic CCA has remained elusive until lately. Athena is intrigued by the recent revelation of a mysterious association between CCA and fetal thyroid function.

In 1987, Greenberg reported a girl with CCA, athelia and mental retardation. [3] During the gestation, her mother had been treated with methimazole and propanolol for hyperthyroidism. Greenberg was quick to suggest that the malformations could be due to the teratogenicity of methimazole. A decade later, another similar case was reported from St.Mary’s Hospital wherein, the victim was a boy and his mother had been exposed to carbimazole - a prodrug of methimazole. [4] Additional case reports concurred with a causal relationship between maternal Graves’ disease (thyrotoxicosis), anti-thyroid medications (especially carbimazole and methimazole) and CCA. [5, 6]

Barwell et al. [7] raised an interesting question whether CCA is attributable to transplacental exchange of maternal auto-antibodies (Thyroid Stimulating Immunoglobulin of Graves disease) or anti-thyroid drugs used in the treatment of thyrotoxicosis. Lee et.al [8] solved the puzzle by studying thyroxin (T4) level in 69 newborns with non-syndromic CCA and 3570 matched controls. They found that fetal T4 levels were inversely related to the risk of CCA. Odds ratio was 0.50 and 0.15 for low and high T4 levels respectively. Thus, suppression of fetal thyroid function, rather than its stimulation, appears to be responsible for CCA. This assumption also corroborates with a report of Cambridge workers who found that mutation of the gene encoding thyroid transcription factor-2 (TTF-2) resulted in thyroid agenesis, CCA and cleft palate in two siblings. [9] All these evidences indicate that fetal hypothyroidism - either due to thyroid agenesis, or due to maternal anti-thyroid therapy - probably causes CCA. This sequela is eventually, albeit somewhat inaccurately, named as “fetal carbimazole syndrome” [10] or “carbimazole embryopathy”. [11] The term “carbimazole embryopathy” is often used interchangeably with “methimazole embryopathy” [12, 13], because the latter drug is simply an active metabolite of the former.

Carbimazole embryopathy occurs when the pregnant woman is exposed to the drug between 1st and 7th week of gestation [13]; especially exposure between 35th and 38th day is critical for the induction of CCA. [6] Carbimazole embryopathy appears to be a spectrum. [14, 15] Approximately 31 cases of carbimazole embryopathy have been reported so far. [16] In addition to CCA (65%) it also includes aplasia cutis (29%), nipple abnormalities (23%), esophageal atresia with- or without- tracheoesophageal fistula (13%), patent vitello-intestinal duct (16%), developmental delay (16%), ventricular septal defects (10%), omphalocoele (6%) and deafness (6%). About 10% of affected embryos develop gastrointestinal anomalies such as Imperforate anus, microcolon, umbilical hernia and gall bladder agenesis. [16]

Until recently the association between carbimazole and CCA has largely been anecdotal, based on isolated case reports. More robust scientific evidence of the association is recently accrued. A multicentric case-control study [17] compared the frequency of prenatal exposure to methimazole in 61 mothers with fetal CCA and 183 control group mothers. Among the 61 cases 10 had exposure to methimazole (16%) as compared to only 2 of the 183 controls (1%); the odds ratio was as high as 17.75. In a further study [18], SAFE-Med study group analyzed 18,131 cases of malformations due to first trimester medications. Prenatal exposure to carbimazole or methimazole in 127 mothers was significantly associated with CCA and omphalocele. 

Agopian et.al [19] studied 280 non-syndromic CCA (including congenital choanal stenosis) and 3720 controls from Texas Birth Defect Registry. They correlated the occurrence of CCA with atrazine levels of the county. Atrazine, a widely used herbicide, was found to increase the risk of CCA by 2 fold. Interestingly, atrazine is a known endocrine disrupter that interferes with maternal thyroxin levels. Atrazine teratogenicity not only explains the occurrence of sporadic CCA but also supports the hypothesis of fetomaternal thyroid dysfunction as the cause of CCA.

CCA of carbimazole embryopathy is frequently accompanied by tracheoesophageal fistula (TEF). Nine cases of carbimazole induced TEF have been reported in the literature. [14,20-23] Interestingly, carbimazole TEF is either atypical or of H-type. Athena contemplates that a thorough understanding of carbimazole embryopathy may unravel the etiopathogenic mysteries of TEF and CCA. She further speculates that fetal hypothyroidism, CCA and TEF could form a new syndrome.

## Footnotes

**Source of Support:** Nil

**Conflict of Interest:** The author is Editor of the journal. But he did not take part in the evaluation or decision making of this manuscript. The manuscript has been independently handled by two other editors.

